# A Mass of Pancreatic and Gastric Heterotopia Causing a Small Bowel Obstruction in a 61-Year-Old Male

**DOI:** 10.1155/2017/3126108

**Published:** 2017-06-01

**Authors:** Majd Alfrejat, Bassem Khalil, Jordan Jackobs, William Anderson, Jennifer Eschbacher

**Affiliations:** ^1^Department of Medicine, St. Joseph's Hospital and Medical Center, Phoenix, AZ, USA; ^2^Department of Medicine, Georgetown University, Washington, DC, USA; ^3^Department of Surgery, St. Joseph's Hospital and Medical Center, Phoenix, AZ, USA; ^4^Department of Pathology, St. Joseph's Hospital and Medical Center, Phoenix, AZ, USA

## Abstract

Heterotopic tissue is a congenital anomaly that has been previously reported. Gastric and pancreatic heterotopia are among the most studied ones. Herein, we describe a case of a combined pancreatic and gastric heterotopia that formed a mass and caused a small intestine obstruction in a 61-year-old male. We also did a brief literature review of cases with gastric and pancreatic heterotopia in adult patients.

## 1. Case Presentation

**Figure 1:**
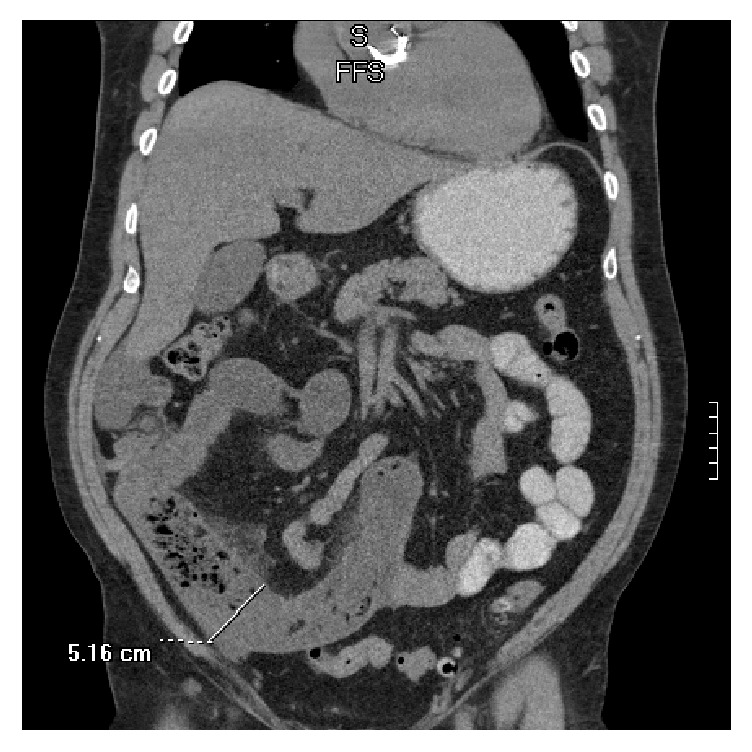
Multiple fluid-filled loops of small bowel with air-fluid levels and dilation most prominently within the right lower quadrant.

61-year-old male with a history of severe aortic valve insufficiency status after prosthetic aortic valve replacement, presented to the emergency department (ED) complaining of epigastric pain, hematemesis, and black stool. The symptoms started one day prior to arrival to the ED. There was no report of dizziness or lightheadedness. Patient was not on any anticoagulation medications. He denied any recent use of Nonsteroidal Anti-Inflammatory Drugs (NSAIDs). On physical exam, his vitals were within normal limits, and he had no orthostatic hypotension. There was abdominal distention, with mild diffuse tenderness. The initial laboratory workup showed a hemoglobin of 12.6 gm/dl with a positive stool guaiac test. The patient underwent an upper endoscopy that revealed three antral ulcers, one of which was clipped. He was also treated with Proton Pump Inhibitor (PPI). He did not require a blood transfusion as his hemoglobin remained stable. The patient continued to report nausea as well as a crampy and occasionally sharp abdominal pain with distention. He also had yellow-colored emesis. An abdominal CT was done and it showed dilated small bowel prominently within the right lower quadrant where it measured 5.2 cm and, immediately proximal to the dilated area, there was fecalization of the small bowel content with wall thickening and mesenteric edema and these findings were consistent with a partial small bowel obstruction (Figure 1).

A surgical consultation was obtained and the patient underwent an exploratory laparotomy for this de novo obstruction. During exploration, he was found to have an obstruction with a clear transition three feet proximal to ileocecal valve. Closer inspection revealed an intraluminal mass associated with the point of obstruction.

**Figure 2:**
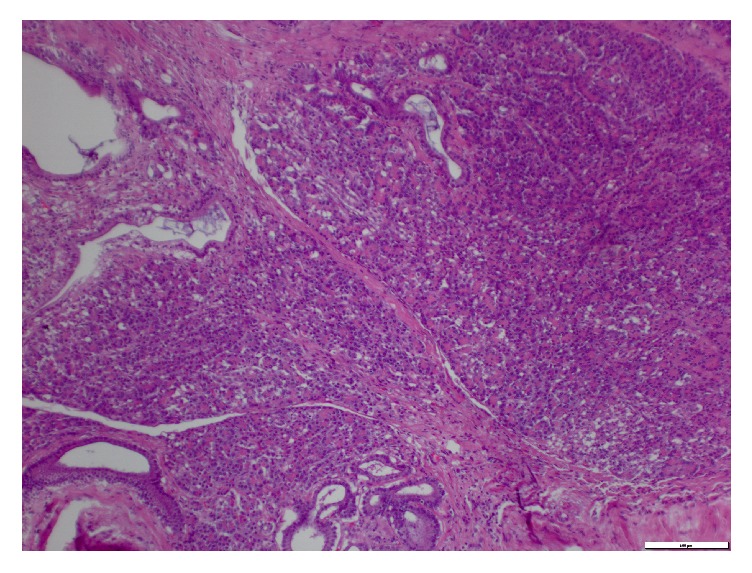
Heterotopic pancreas (100x).

**Figure 3:**
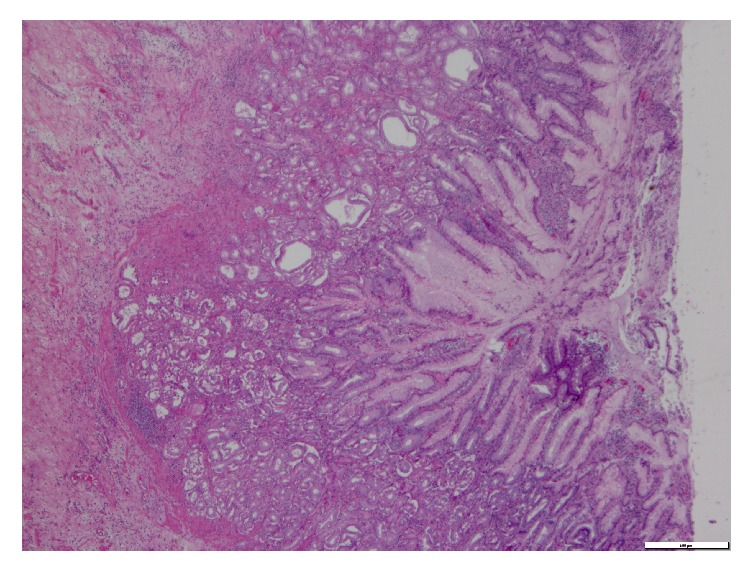
Heterotopic gastric tissue (40x).

There was no diverticulum in the area. A segmental small bowel resection with an enteroenterostomy was completed. The mass measured 3.0 × 2.5 × 2.5 cm, and it contained a dense tan focally necrotic cut surface that grossly abutted the serosal border without extending into the mesenteric fat. The microscopic pathological examination revealed heterotopic pancreas, heterotopic gastric tissue, and an isolated benign mesenteric lymph node with no diverticular tissue (Figures 2 and 3).

The patient had a short and complications-free postoperative course. He was tolerating regular diet in the third day following the surgery and was discharged home in a stable condition.

## 2. Discussion

The word “heterotopia” is derived from Greek and it means “other places.” Heterotopic tissue is a congenital anomaly defined as the presence of the tissue outside its usual location.

The earliest report of heterotopic pancreas (HP) was done without the aid of microscopic confirmation by Jean Shultz in 1727. Nevertheless, Klop was the first one to describe it in 1859 [1]. Heterotopic gastric mucosa (HGM) was first described by Schmidt in 1805 when he reported a case of HGM in the esophagus [2].

The most generally agreed-upon mechanism to explain the pathogenesis of HP was suggested by Hogan who proposed that it could be due to an early attachment of small pancreatic buds to the intestinal wall at different sites, and when this connection continues, even after the pancreas separates from the gut, it results in HP [3]. As for the HGM, there are three etiologies that were suggested to explain this condition. It could occur due to a growth error during the descent of the stomach, when few cells that are designed to form part the gastric mucosa fail to descend and end up remaining in the esophagus [4]; or could be due to a differentiation error. when some of the cells in the undeveloped intestinal canal, that have broad differentiation characteristics, develop into a gastric epithelium at an incorrect level [5]; or lastly could be due to a regeneration error following the eradication of preexisting mucosa as seen in cases of regional enteritis and chronic tuberculous ulcer of the colon [6, 7].

While both heterotopias could occur at any site of the GI tract, the stomach, the duodenum, and the upper part of the jejunum were found to be the most common locations of HP [8]. The estimated incidence of HP ranges between 0.55% and 13.7% in autopsy studies and is one in every 500 upper abdominal laparotomies [9, 10]. One the other hand, HGM is the most reported epithelial heterotopia [11]. It is particularly found in the esophagus, duodenum, and ileum (Meckel's diverticulum) [12], with widely variable range of prevalence estimated from 0.1% atond 13.8% in the esophagus [13, 14] and 0.5% to 8.9% in the duodenum [15, 16].

The clinical manifestations depend on the site where the heterotopia is found.

In one of the largest retrospective studies that was done on HP, Dolan et al. reviewed the chart of 212 patients who were diagnosed with HP at the Mayo Clinic between 1952 and 1971. The most common presenting symptom was abdominal pain followed by melena, diarrhea, gas, and bloating. The study did not find a relation between the surgical intervention and the resolution of the presenting symptom except in the case of the GI blood loss [17]. Furthermore, HGM was reviewed by Wolff in a study of 87 cases that involved the entire GI tract. Aside from Meckel's diverticula, the highest number of HGMs was associated with regional enteritis followed by peptic ulcer disease [18]. It can also cause more rare manifestations such as painless rectal bleeding when it is located in the large bowel and cholecystitis when found in the gallbladder [19].

To the best of our knowledge, there were only 4 other cases in the literature that reported a combined heterotopia of pancreatic and gastric tissue [20–23]. In two of these cases, the heterotopic tissues were found in the large bowel and manifested as a diverticulum of the transverse colon in the first case [20] and an ulcerated mass at the splenic flexure that caused a lower gastrointestinal bleed in the second case [21]. In the third report, the combined heterotopic tissues were found incidentally in the gall bladder following a cholecystectomy [22], whereas, in the fourth and the most recently published case, the patient had an obstructive ileal tumor that contained HP, HGM with submucosal lipoma, and caused chronic abdominal pain [23]. In our case, the symptoms resolved after the surgical resection of the heterotopic tissues.

## 3. Conclusion

Although it is not very common, heterotopic tissue should be always considered in the differential diagnosis of adult patients presenting with a common digestive complaint.
