# Corrigendum to “A Mass of Pancreatic and Gastric Heterotopia Causing a Small Bowel Obstruction in a 61-Year-Old Male”

**DOI:** 10.1155/2017/2536163

**Published:** 2017-09-24

**Authors:** Majd Alfreijat, Bassem Khalil, Jordan Jackobs, William Anderson, Jennifer Eschbacher

**Affiliations:** ^1^Department of Medicine, St. Joseph's Hospital and Medical Center, Phoenix, AZ, USA; ^2^Department of Medicine, Georgetown University, Washington, DC, USA; ^3^Department of Surgery, St. Joseph's Hospital and Medical Center, Phoenix, AZ, USA; ^4^Department of Pathology, St. Joseph's Hospital and Medical Center, Phoenix, AZ, USA

In the article titled “A Mass of Pancreatic and Gastric Heterotopia Causing a Small Bowel Obstruction in a 61-Year-Old Male” [1], the name of the first author was given incorrectly as Alfrejat. The author's name should have been written as Alfreijat. The revised authors' list is shown above.
